# Development and Proof of Concept of a Low-Cost Ultrasound Training Model for Diagnosis of Giant Cell Arteritis Using 3D Printing

**DOI:** 10.3390/diagnostics11061106

**Published:** 2021-06-17

**Authors:** Florian Recker, Lei Jin, Patrick Veith, Mark Lauterbach, Pantelis Karakostas, Valentin Sebastian Schäfer

**Affiliations:** 1 Department of Obstetrics and Prenatal Medicine, University Hospital Bonn, 53127 Bonn, Germany; florian.recker@ukbonn.de; 2 Clinic of Internal Medicine III, Department of Oncology, Hematology, Rheumatology and Clinical Immunology, University Hospital Bonn, 53127 Bonn, Germany; leijin1987@hotmail.com (L.J.); pantelis.karakostas@ukbonn.de (P.K.); 3 Hochschulrechenzentrum, University Bonn, 53115 Bonn, Germany; pveith@uni-bonn.de (P.V.); Lauterb@uni-bonn.de (M.L.)

**Keywords:** giant cell arteritis, model development, diagnosis, ultrasound, 3D printing

## Abstract

Objectives: Currently, ultrasound (US) is widely used for the diagnosis of giant cell arteritis (GCA). Our aim was to develop a low-cost US training model for diagnosis of GCA of the temporal and axillary artery using a modern 3D printing system. Methods: We designed an US training model, which enables measurement of the intima-media thickness (IMT) of temporal and axillary arteries using Autodesk Fusion360. This model was printed using a modern 3D printer (Formlabs Form3) and embedded in ballistic gelatine. The ultrasound images including measurement of the IMT by ultrasound specialists in GCA were compared to ultrasound images in acute GCA and healthy subjects. Results: Our ultrasound training model of the axillary and temporal artery displayed a very similar ultrasound morphology compared to real US images and fulfilled the OMERACT ultrasound definitions of normal and pathological temporal and axillary arteries in GCA. The IMT measurements were in line with published cut-off values for normal and pathological IMT values in GCA and healthy individuals. When testing the models on blinded US specialists in GCA, they were identified correctly in all test rounds with an intra-class coefficient of 0.99. Conclusion: The production of low-cost ultrasound training models of normal and pathological temporal and axillary arteries in GCA, which fulfil the OMERACT ultrasound definitions and adhere to the published IMT cut-off values in GCA, is feasible. Ultrasound specialists identified each respective model correctly in every case.

## 1. Introduction

Giant cell arteritis (GCA) is a systemic autoimmune disease and the most common form of vasculitis in adults over 50 years of age, characterized by the inflammation of medium- and large-sized arteries. The temporal and axillary arteries are commonly involved. Early changes consist of transmural inflammation of the arterial wall, while later complications include lumen changes, such as stenosis or aneurysms [1]. A rapid diagnosis and treatment are important in order to prevent serious vascular complications such as blindness and other vascular events [2]. The current recommendations by European League Against Rheumatism (EULAR) highlighted the diagnostic value of ultrasound of the temporal and axillary arteries in GCA [3]. Ultrasound is commonly available in rheumatology practice and is patient-friendly, reproducible, and repeatable [4]. For diagnosis of GCA, the OMERACT ultrasound definitions and the respective cut-off values for normal and pathological temporal and axillary arteries are used [5]. Several studies showed that ultrasound training with novice sonographers resulted in excellent ultrasound reliability in patients suspected of GCA [6,7]. Thus, the role of ultrasound in training and education in GCA is crucial. Since every single GCA case is unique and without appropriate training models that are realistic and long lasting, practicing these skills can prove to be difficult, which poses a huge challenge for rheumatologists worldwide.

Three-dimensional (3D) printing is an emerging technology that builds up a physical model commonly in a layer-by-layer manner using a 3D computerized model reconstructed from a series of images such as computed tomography (CT) or magnetic resonance imaging (MRI) datasets [8,9,10]. In clinical practice, 3D printing is reported to have been used in a variety of subjects. The most striking usage is to study complex cases and practice procedures in medical education [11,12,13]. Fused deposition modelling (FDM) and stereo-lithography (SLA) 3D printing technique is a promising method for making vessel-mimicking phantoms (VMPs) with a complex vessel lumen [14,15]. Theoretically, 3D printed models have better spatial and structural visualization and can be used as didactic tools for better understanding of vessel wall pathologies in GCA, including increased intima-media thickness (IMT), aneurysms and vascular stenosis [11,16]. However, there is no rheumatology ultrasound vasculitis model for GCA available to date, which could be used for teaching purposes.

In this study, we aimed to establish a low-cost 3D-printed ultrasound training model of a normal and pathological temporal and axillary artery in GCA, fulfilling the OMERACT ultrasound definitions for GCA and the published cut-off values of the IMT.

## 2. Materials and Methods

The process of producing the final model is described in the following next three steps.

### 2.1. Step 1—Creating Three-Dimensional Models of the Temporal and Axillary Artery

**Figure 1:**
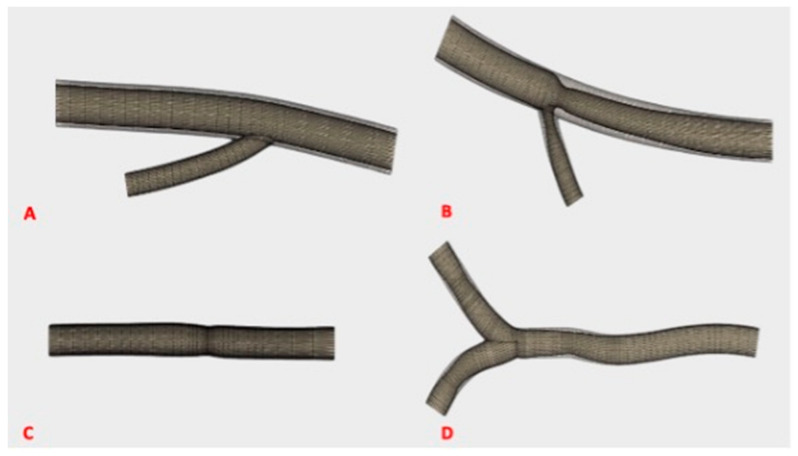
Digital model of a normal and pathological axillary artery. (**A**) Model of a normal axillary artery. (**B**) Model of a pathological axillary artery in giant cell arteritis with an increased intima-media complex. (**C**) Model of a normal temporal artery. (**D**) Model of a pathological temporal artery in giant cell arteritis with increased intima-media complex.

Based on ultrasound images of patients, the published OMERACT ultrasound definitions of normal and pathological axillary and temporal arteries in GCA and the published cut-off values [5], artery models were designed and converted using Fusion360TM (Autodesk Inc., San Rafael, CA, USA) into a stereo-lithography (.stl) file (Figure 1).

### 2.2. Step 2—Printing Process

We used the Formlabs Form3^®^, a low-force Stereo-lithography^tm^ based 3D printer (Formlabs Inc., Somerville, MA, USA), which uses liquid flexible resin incorporated as print material. In this way, the print resolution increased to 50 µM per line. However, the program was more complex than FDM and included a chemical reaction of the resin under laser light instead of merely melding the PLA. It also comprised of a step to wash the prints in isopropanol alcohol in a form wash station and a step to harden the material in a form cure station with 405 nm Light-emitting diode (LED) light. We used black resin from Formlabs^®^ Inc. and this resulted in a possible resolution of 0.05 mm up to 0.025 mm, which produced less interpolation during the print process. The intended artery wall thickness did result in prints, but the weight of the resin of the model in large prints started to deform itself as each layer is pulled from the transparent surface at the bottom of the resin tank during the printing process. The differences between the various resins lie on the one hand in their heat stability and on the other hand in the overall stability of the printing material used. In addition, each resin displayed a different echogenicity.

Thus, we had to use support structures to reduce these deformations. Furthermore, the chemical printing process, the isopropanol bath, the LED-light, and the 60 °C heat hardening process put additional stress on the prints. In addition to the software settings for the print, the low-force Stereo-lithography^tm^ printing process introduced new influencing factors for the prints, which had to be considered.

**Figure 2:**
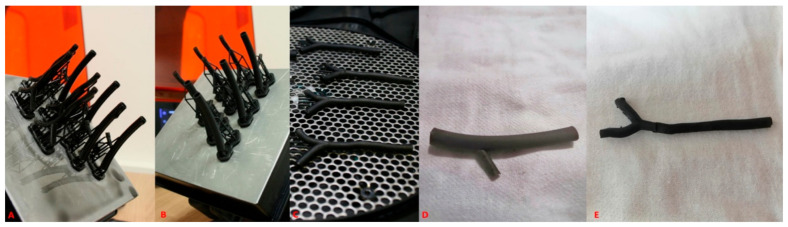
Demonstration of the printed models of the temporal artery with the low-force Stereo-lithography^tm^ technique and supporting structures. Manufacturing process of the temporal artery models at various stages. (**A**) Picture taken just after the printing process finished of an axillary artery model. (**B**) Image after washing of the axillary artery models in isopropanol alcohol. The black resin is much less brittle after the hardening process, so the supports were cut away after the washing process. (**C**) Picture after hardening of temporal artery models with 405 nm LED light. (**D**,**E**) high resolution images of a temporal and axillary model respectively.

In contrast to the FDM printer, where we could not easily influence the way each layer was printed, we were forced to design model specific support structures, which did not interfere with the ultrasound purpose of the prints in this new printing process (Figure 2).

### 2.3. Step 3—Embedding of the Model in Gelatine

In order to allow a realistic ultrasound experience and correlate with real ultrasound images, we embedded our 3D models into a specific clear ballistic gelatine. Ballistic gelatine is a testing medium scientifically correlated to swine muscle tissue (comparable to human muscle tissue), with similar ultrasound propagation velocity as that of human connective tissue.

**Figure 3:**
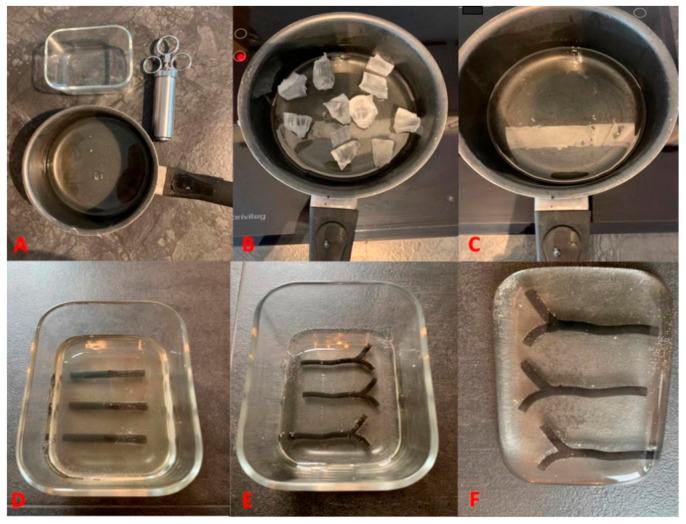
Embedding procedure of the printed model into ballistic gelatine. (**A**) Materials needed (oven-safe glassware, melting pot, one pound of gelatine. and injection syringe for filling of vessel lumen), (**B**) gelatine melting process, (**C**) melted gelatine, (**D**) embedding of the printed models into the gelatine, (**E**) hardening process of the gelatine, (**F**) final artery model.

Embedding the models into the gelatine was performed as follows. First, the oven-safe glassware, an oven and one pound of gelatine were prepared (Figure 3A). Next, the gelatine was obtained from the manufacturer in square blocks of 41 cm × 15 cm × 15 cm for EUR 204.50. The blocks of ballistic gelatine were cut into 1 cm × 1 cm cubes to decrease melting time (Figure 3B). Thereafter, the oven was set to 130 °C, and the oven-safe glassware containing cubed pieces was placed into it. The material was checked regularly until it has melted completely, approximately requiring one hour (Figure 3C). Finally, the melted gelatine was poured into the container, and the vessel models with different wall thickness were placed around 1 cm underneath the gel surface (Figure 3D). The gelatine was then cooled. Once the material hardened, the gelatine model was removed from the glassware (Figure 3E). Reusing the gelatine is relatively easy. The embedded model is broken out of the gelatine. Then the remaining gelatine residue can simply be reheated in a pot and is reboiled into a liquid mass. After that, it can be reused to embed structures. Even after multiple boiling and embedding processes (>20 times), the gelatine did not change its properties.

### 2.4. Ultrasound Examination of the 3D-Printed Models

All ultrasound examinations were performed with an Esaote MyLab Twice eHD ultrasound machine built in 2014. We used the same ultrasound technique as described by the OMERACT ultrasound group on large vessel vasculitis [4]. The following settings were applied for the examination of the temporal arteries (axillary arteries): B-mode frequency 18 MHz (14 MHz), image depth 1.5 cm (3 cm), and 1 focus point at 0.5 cm (1.5 cm) below skin surface. For the common superficial temporal artery parallel to the surface, IMT measurements were performed. In the transversal and longitudinal sections, measurements were carried out in mm on the vessel wall distal to the probe. We used the OMERACT ultrasound definitions as described before [5]. Two ultrasound specialists (PK and VSS) in diagnosis of GCA examined each model of a normal and pathological temporal and axillary artery including measurement of the IMT. The sonographers were blinded to inspect whether the model of the respective artery was pathological or not. We tested four models five times. Examination was repeated after 48 h for determination of intra-rater reliability. Examiners had to choose if the respective model displayed a normal or pathological temporal or axillary artery and perform an IMT measurement at the most prominent site. The vessel wall pathology of the axillary and temporal artery could not be identified from the outside of the model, as the changes were restricted to the intima of the vessel, nor were the models somehow distinguishable.

### 2.5. Statistical Analysis

Statistical analysis was performed with R statistical software (v.i386 4.0.2). Means and standard deviations were calculated as descriptive parameters. Inter-rater agreement was calculated using intra class coefficient (ICC) (2.1) between the readers. The ICC were interpreted according to Rosner [17]: ICC < 0.4 indicated poor reliability, 0.4 ≤ ICC < 0.75 fair to good reliability, and ICC ≥ 0.75 excellent reliability. Bland–Altman plots were designed with StataIC v.16.1 software (StataCorp, Lakeway, TX, USA) [18]. For quantitative parameters, the mean SD and range were determined. Significant changes were calculated by using *t*-test and Spearman correlation [19].

## 3. Results

**Figure 4:**
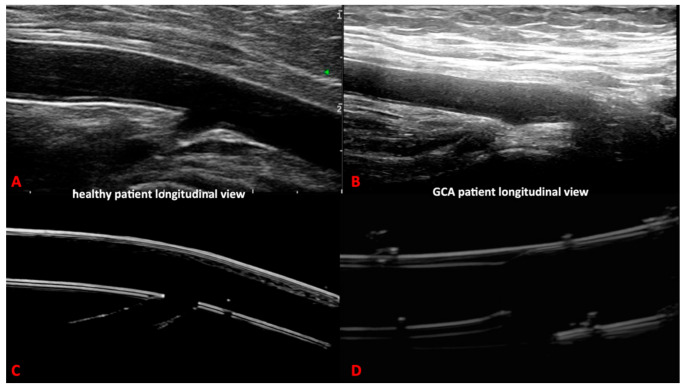
Comparison between ultrasound images of the axillary artery of the 3D printed models (**C**,**D**) and real ultrasound images in healthy and giant cell arteritis patients (**A**,**B**).

**Figure 5:**
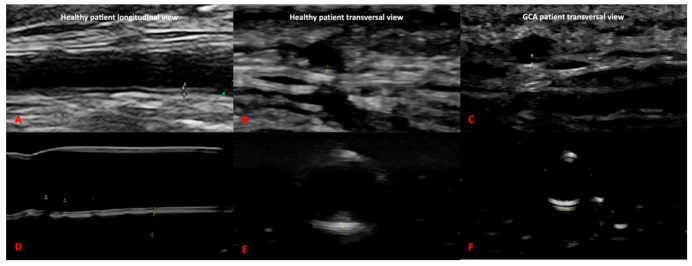
Comparison between ultrasound images of the temporal artery of the 3D printed models of healthy (**D**,**E**) and pathological setting (**F**) and real ultrasound images in healthy (**A**,**B**) and giant cell arteritis patients (**C**).

Our 3D printed artery models fulfilled the OMERACT definitions of normal and pathological temporal and axillary arteries in GCA [5] (Figure 4 and Figure 5). In addition, we were able to replicate the IMT of the corresponding arteries, allowing the measurement of the IMT values of the normal and pathological arteries. These values corresponded to the published cut-off values [20,21,22] for ultrasound diagnosis in GCA of the temporal or axillary arteries.

### 3.1. Identification of Each 3D-Printed Artery Model

**Figure 6:**
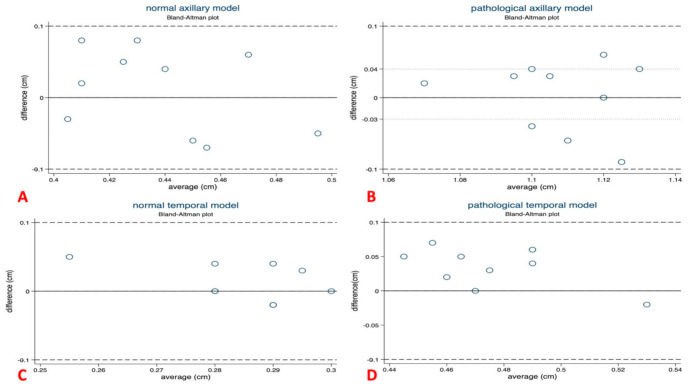
Bland–Altman plots on reliability of the four different US models. (**A**) Bland–Altman-plot of the normal axillary model (*p* = 0.536); (**B**) Bland–Altman-plot of the pathological axillary model (*p* = 0.853); (**C**) Bland–Altman-plot of the normal temporal model (*p* = 0.280); (**D**) Bland–Altman-plot of the pathological temporal model (*p* = 0.003). Overall range of intima-media thickness measurements was good to excellent in all four models.

The two ultrasound specialists examined the four different models five times each on two different days. Correct identification of the pathological and physiological temporal and axillary arteries was achieved by both examiners in all examination rounds. Consequently, the detection based on the vessel models was 100% correct. The total averaged measured values of the four different models were as follows: for the normal axillary model, 0.44 mm (SD ± 0.30), and for the pathological axillary model, 1.11 mm (SD ± 0.34). Further, the data were, for the model of the normal temporal artery, 0.27 mm (SD ± 0.31), and for the pathological, v.0.48 mm (SD ± 0.30). When testing the reliability of the four different models, the two independent raters (PK and VSS) showed an inter-rater reliability (ICC 2.1) of 0.99 (95% interval 0.98 < ICC < 0.995) in detecting pathological vs. normal temporal and axillary artery models. Comparing the IMT measurements, the two raters displayed a strong degree of agreement between the different measurements. There were no cases in all four models where the two raters made measurements outside the standard deviation (Figure 6) [18].

Applying the OMERACT definitions for normal and pathological temporal and axillary arteries, the sonographers made the correct diagnosis in every round and every model. Furthermore, all four models were shown to be suitable for the simulation and training of sonographic detection of GCA.

### 3.2. Cost Effectiveness

A single model print took approximately nine hours and used 2.8 mL of resin, costing approximately EUR 0.45 and approximately EUR 1.20 for electricity used during printing, yielding a total of EUR 1.65 per model. The printer took about 19 h to complete 12 prints in total. Additionally, we used some isopropanol, which can be reused many times, and extra electricity for washing and hardening. The cost of the gelatine was EUR 4.08 per model, resulting in a total price of EUR 5.28 per model.

Of course, additional costs such as depreciation of the 3D printer and personnel costs have to be considered as well; these are dependent on the 3D printer as well as country-specific wages.

## 4. Discussion

Ultrasound can be performed simultaneously with recording history of the patient and clinical examination by the clinician and is widely used in European countries by now. It is patient-friendly, reproducible, and repeatable. It can be used in fast-track clinics offering appointments for patients within 24 h, to rapidly confirm or exclude the diagnosis of suspected GCA [23].

When training a physician in ultrasound in suspected GCA, the most common problem is the number of patients the physician has to examine before he is experienced in ultrasound diagnosis of GCA. Failure to accurately diagnose and expeditiously treat GCA may lead to vision loss and other severe ischemic complications, whereas misdiagnosis of non-GCA pathology as GCA leads to inappropriate glucocorticoid use and toxicity.

Thus far, there is no ultrasound model in the literature available for training and teaching purposes regarding the ultrasound diagnosis of GCA. In this first probability study, we were able to demonstrate the feasibility and reliability of a low-cost 3D printed vessel model of a normal and pathological temporal and axillary artery in GCA.

Designing a suitable vessel model and finding the perfect gelatine did take us a total of 12 months, as we struggled with different materials and 3D printers. However, we were finally able to 3D print a standardized artery model of the axillary and temporal artery, with either normal or pathological IMT changes fulfilling the OMERACT definitions [5] and displaying typical IMT changes [23]. When we tested the four models on two experienced sonographers in vascular ultrasound in GCA, the intra-class coefficient of normal and pathological temporal and axillary arteries in GCA were excellent for the diagnosis of GCA.

Compared to conventional ultrasonic coupling agents, this study had the following innovations. The components of the model described in this paper are unique when compared with other materials used to create models. It is unlike agar models, which need to be refrigerated, and it does not tear easily. Furthermore, our material does not require mixing with other agents such as fibre, gauze, and sawdust to create tissue like densities. Our gelatine does not dry out or decay, and it is reusable. The material is clear which allows trainees to have direct visual access to the vessel. The ultrasound training models displayed a very similar ultrasound morphology compared to real ultrasound images and fulfilled the OMERACT ultrasound definitions of normal and vasculitis of temporal and axillary arteries during the two exercises.

Of course, every single GCA case is unique, and in the present study, we only designed one model version for each, normal and pathological, of the temporal and axillary artery each. First, this is a pilot study to verify its feasibility in design and production of a training model for vascular ultrasound in GCA. Second, we thought it was enough to use only temporal and axillary artery models for medical education purposes, as these are the most frequently examined arteries in suspected GCA [3]. We received exceptional results after testing our model with two highly skilled sonographers. Every model version of the axillary and temporal artery was identified correctly as normal or pathological. Further, the IMT measurements were reproducible and reliable, helping in diagnosis. We propose to study this model in a larger cohort of ultrasound specialists in GCA, which is planned for 2021 within the OMERACT ultrasound large vessel vasculitis group.

However, there were some limitations. Firstly, although the printing materials of the vasculitis model were cheap, with about EUR 5.28 per model, the cost of 3D-printed parts depended heavily on the manufacturing facility and the resin used. Therefore, it could be cost effective to print more models in the long run. Cheap desktop 3D-printers allow cheaper 3D models but have fewer quality approvals and controls than commercial manufacturers, who are required to meet high quality standards, like the printer we used [11]. We used Formlabs Form3^®^, an excellent material for 3D printing of medical materials that is supplied with the form cure and wash station and manufactured resin without ever being touched. Secondly, the models were fragile after the hardening process and easy to break by compression as the training of “compression” signs cannot be carried out. Thirdly, these 3D prints are not without drawbacks. Several factors such as the path for the laser and the chemical process of hardening resin with the laser impacted the precision of the print. It remains a challenge to accurately print out vascular structures; however, we were able to reproduce many models with very similar IMT measurements. This may however differ when other printers are used.

## 5. Conclusions

We were able to produce a low-cost 3D model of normal and pathological temporal and axillary arteries in GCA with a price of EUR 5.28. In the near future, we plan to test our models in a greater cohort of ultrasound specialists and further work on the resolution of the vessel model as well as produce different versions in order to offer a wider range of pathologies.
